# Epitope Spreading: The Underlying Mechanism for Combined Membranous Lupus Nephritis and Anti-GBM Disease?

**DOI:** 10.1155/2023/3190042

**Published:** 2023-01-24

**Authors:** Olusola Sogbein, Tina Kochar, Marjan Afrouzian

**Affiliations:** ^1^Department of Internal Medicine, Division of Nephrology and Hypertension, University of Texas Medical Branch, Galveston, Texas, USA; ^2^Department of Pathology, University of Texas Medical Branch, Galveston, Texas, USA

## Abstract

Membranous lupus nephritis associated with anti-GBM antibodies is a rare entity, particularly in lupus nephritis patients who are serologically negative for ANA and anti-dsDNA with normal complement levels. We present an unusual case of a patient initially diagnosed with anti-GBM disease whose repeat biopsy demonstrated combined focal proliferative and membranous lupus nephritis (III + V). The first biopsy showed a granular linear pattern, and the second biopsy had multiple electron dense deposits in the subendothelial, epithelial, and mesangial regions along with podocyte effacement. Experimental research suggests that the sequential histopathological transition observed may reflect the action of immunological rearrangement and epitope spreading.

## 1. Introduction

Systemic lupus erythematosus (SLE), the prototype of all autoimmune diseases, involves the kidney in approximately 50%–90% of cases [1]. Lupus nephritis (LN) is accompanied by significant mortality and morbidity, particularly when associated with rapidly progressive glomerulonephritis (RPGN). The vast majority of patients with LN are positive for antineutrophil antibody (ANA) and antidouble stranded DNA (dsDNA) [1, 2]. Therefore, positive serology is included in the American College of Rheumatology LN Criteria [3]. However, up to 5% of patients with LN can be seronegative, requiring diagnosis based on supportive criteria [4]. In addition, there are scattered case reports of LN patients presenting with antiglomerular basement membrane (anti-GBM) antibodies. Experimental evidence supports the role of the kallikrein gene as a lupus-susceptibility factor predisposing certain patients to LN and anti-GBM disease [5–7].

Classically, the pathological presentation on immunofluorescence (IF) involves the presence of IgG, IgA, IgM, Kappa, and Lambda, in addition to complement pathway deposits (−C3, C4, and C1q)—the “full-house pattern” [8–10]. Recently, a rare number of cases with crescentic formation associated with linear IgG deposition on IF consistent with anti-GBM disease and subsequent conversion to membranous glomerulopathy (MGp) associated with granular deposits have been reported [11–14]. We present a rare case of seronegative lupus nephritis with serologic positivity for anti-GBM antibodies (anti-GBM Ab) and pathological evidence of membranous lupus nephritis (MLN), Class V.

## 2. Case

**Figure 1:**
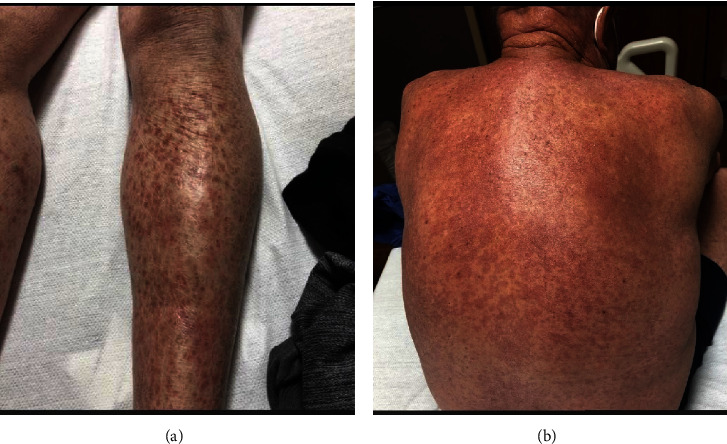
(a) Bilateral purpura of the limbs demonstrating small-vessel vasculitis. (b) Purpura of the posterior thoracic and lumbar regions.

A 62-year-old Caucasian male presented with pulmonary nodules and a year-long history of rash and tea-colored urine. The rash started in his lower extremities and spread to his upper extremities and back (Figures 1(a) and 1(b)). There were no other associated symptoms such as pruritus, fatigue, or joint pain. His laboratory tests revealed a creatinine of 2.2 mg/dL, white blood cell count of 8.3 × 10^3^ *μ*L, hemoglobin of 12.8 g/dL, platelet count of 342 × 10^3^ *μ*L, normal liver function tests, and D-dimer of 1770 mg/mL. He was diagnosed with a prerenal acute kidney injury and started on intravenous fluids. A week later, the patient experienced an episode of severe vertigo, nausea, and vomiting and was taken to the emergency department. Urinalysis showed amber colored urine with 500 mg/dL of protein and 182 RBC/HPF with 18 WBC/HPF. Urine microscopy showed mostly eumorphic RBCs. Over the next 48 hours, his creatinine increased from 2.35 to 3.14, and a renal biopsy was performed. The patient's protein/creatinine ratio (PCR) was 2.3 g/g, complement levels were low (C3−: 39 and C4−: 2), urine and serum electrophoresis were unremarkable, cryoglobulins were absent, ANA titer was 1 : 20, and anti-dsDNA was 3.0 IU/mL. Further, his IgG antiglomerular basement membrane antibody level by multiplex bead assay was reported at a level of 63 AU/mL (normal 0–19).

**Figure 2:**
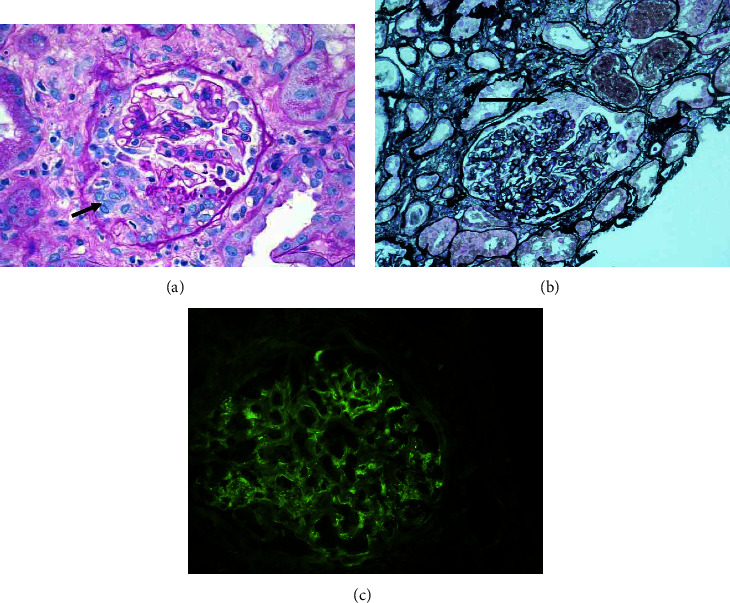
(a) First renal biopsy (LM): a glomerulus containing a cellular crescent (PAS, ×400). (b) First renal biopsy (LM): cellular crescent without evidence of hypercellularity (arrow) (PAMS; ×200). (c) First biopsy: deposition of granular to segmental immune complexes along the GBM (IF, IgG; ×400).

The initial renal biopsy was inadequate. By light microscopy (LM), up to 4 glomeruli were present. None of the glomeruli were globally sclerotic. Two glomeruli contained cellular crescents (Figures 2(a) and 2(b)). Peripheral capillary loops were patent, and mild endocapillary proliferation was present in a few segments of two glomeruli. The endocapillary proliferation was mainly composed of neutrophils and enlarged endothelial cells. A mild increase in mesangial matrix and cellularity was also seen. The glomerular basement membrane (GBM) appeared mildly collapsed adjacent to the crescent; no spikes or holes were visible on the silver stain, and capillary microthrombi or tuft necrosis was absent. The tubules showed evidence of acute tubular injury and necrosis and contained cellular debris and red blood cell casts. There were extensive nuclear regenerative changes. Interstitial fibrosis and tubular atrophy were of moderate degree. The interstitium was infiltrated by a mixed infiltrate, and peritubular capillaritis was present in some areas. Arteries showed mild fibrous intimal thickening, and arterioles were hyperplastic. By immunofluorescence microscopy (IF), immune complex deposits were present along the GBM with the following intensities: IgG (+++); IgA (trace); C3/C1q/Kappa/Lambda (++). The deposits had a peculiar pattern and appeared granular along some capillary loops but revealed a segmental, linear pattern in other areas. However, typical ultra-linear deposits of IgG, as seen in anti-GBM disease, were not observed (Figure 2(c)). No tissue was submitted for electron microscopy (EM). Therefore, the first biopsy was judged as inadequate but suspicious for anti-GBM disease due to the segmental and linear character of the deposits.

**Figure 3:**
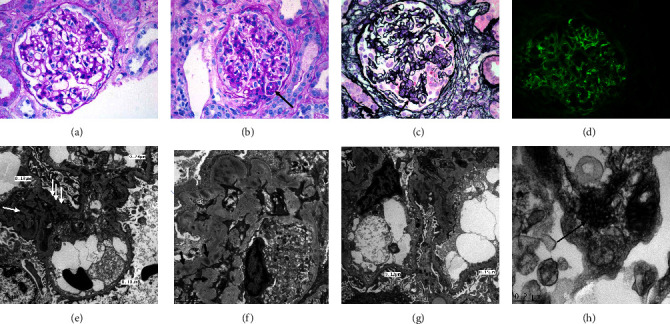
(a) Second biopsy: a glomerulus with no evidence of hypercellularity (PAS; ×400). (b) Second biopsy: a glomerulus with mild endocapillary proliferation (arrow) (PAS; ×400). (c) Secondary biopsy: a glomerulus with endocapillary proliferation in two segments (arrows) and, GBM breaks and crescent formation between the two segments (PAMS; ×400). (d) Secondary biopsy: deposition of granular immune complexes along the GBM and, to a lesser degree, within the mesangium (IF, IgG; ×400). (e) Second biopsy: thin GBM, intramembranous and para-mesangial electron dense deposits (arrows), and focal effacement of foot processes (EM; scale bar = 2 *μ*). (f) Second biopsy: intramembranous and mesangial electron dense deposits (arrows), and effacement of foot processes (EM; scale bar = 2 *μ*). (g) Second biopsy: thin GBM, intramembranous and para-mesangial electron dense deposits, endothelial cell swelling and loss of fenestration (EM; scale bar = 2 *μ*). (h) Tubulo-reticular inclusions within the endothelial cytoplasm (EM; scale bar = 22 *μ*).

Due to the rapidly worsening renal function, positive anti-GBM serology, and initial biopsy results, the patient was started on cyclophosphamide and corticosteroids and underwent one session of therapeutic plasma exchange (TPE). A second kidney biopsy was performed 48 hours later. By LM, twenty-two glomeruli were present, five of which were globally sclerotic. The remaining glomeruli showed a spectrum of lesions: the majority of the glomeruli appeared normal, with patent capillaries and no evidence of proliferation (Figure 3(a)). A minority of glomeruli showed one or two mesangial areas with more than 4 nuclei (mild mesangial proliferation). Three glomeruli revealed endocapillary proliferation (Figures 3(b) and 3(c)) with the presence of rare neutrophils, and three glomeruli showed small, noncircumferential cellular crescents. One glomerulus contained GBM breaks by silver stain and small spikes and a few double contours were depicted in other glomeruli. No necrosis, intracapillary microthrombi, or cryoglobulins were observed. The tubules showed signs of acute tubular injury and evidence of regeneration. The interstitium was infiltrated by lymphocytes and rare neutrophils. Some tubules contain red blood cell casts. Interstitial fibrosis and tubular atrophy were of mild degrees, involving 10% of the parenchyma. The arteries showed moderate fibrous intimal thickening and duplication of the elastic lamina, and the arterioles were hyperplastic. By IF, this time, the pattern of immune complex deposition was clearly granular (Figure 3(d)) and observed along the GBM and within the mesangium with the following intensities: IgG/C3/C1q (+++); IgA/Kappa/Lambda (++); and IgM (+). By EM, the GBM appeared uniformly thin, with an average thickness of 170.5 nm. Mesangial interposition is observed in many capillary loops. Multiple small and large electron dense deposits were found in the subendothelial, intramembranous, subepithelial, and mesangial regions. Tubulo-reticular inclusions were also present in the endothelial cytoplasm, and foot processes were effaced in many areas (Figures 3(e)–3(g)). Multiple tubulo-reticular inclusions were present within the endothelial cytoplasm (Figure 3(h)).

Based on the presence of mesangial and endocapillary proliferation, double contours, full-house pattern of immune complex deposition, especially C1q, and the presence of electron dense deposits and tubule-reticular inclusions in the second biopsy, as well as the immunofluorescence pattern in the skin biopsy, a diagnosis of combined focal proliferative and membranous lupus nephritis (III + V) was rendered.

**Figure 4:**
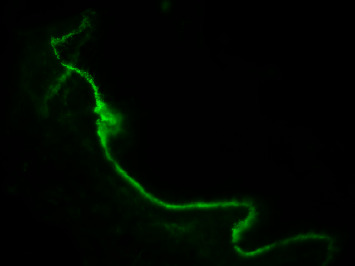
Skin biopsy. Lupus band: linear immune complexes deposition along dermo-epidermal junction (IF, C1q; ×400).

A skin biopsy revealed the presence of the lupus band with deposits of IgG/IgA/IgM/C3/C1q at the dermo-epidermal junction, confirming the diagnosis of SLE (Figure 4).

The patient received a final diagnosis of combined membranous and focal lupus nephritis with associated serum anti-GBM antibodies. Neither complement levels nor anti-GBM antibody titers were repeated. He began induction therapy with mycophenolate 500 mg twice daily, which was gradually increased to 3 grams daily in addition to prednisone (1 mg/kg). He responded well, with a decrease in UPCR from 2.3 to 0.3 over the next four months.

## 3. Discussion

Our knowledge about SLE and LN has evolved greatly during the second half of the 20^th^ century. The exaggerated B-cell and T-cell response and the absence of adequate tolerance against self-antigens are at the core of the pathophysiology of tissue destruction in this interesting autoimmune disease [15]. When the kidney is involved, excessive biosynthesis of antibodies, glomerular immune complex deposition, complement, and cytokine activation are the most important factors that contribute to the lesions observed in LN [15]. On the other hand, anti-GBM antibody deposition consists of a continuum of diseases ranging from renal limited involvement (anti-GBM disease) to pulmonary-renal syndrome with alveolar hemorrhage and RPGN [16–18]. Anti-GBM disease is a rare disease, occurring in 0.5–2 cases/million/year [19]. Due to the paucity of serological data on anti-GBM-positive subjects, the prevalence of LN with associated anti-GBM antibodies has not been defined.

A study from China reports on 14 out of 157 LN patients with anti-GBM antibodies [7]. The authors speculate that immune complex deposition within the GBM leads to activation of the complement cascade and an increase in the release of vasoactive substances that cause glomerular damage [7]. Sugawara et al. report on a case of anti-GBM disease accompanied by LN and discuss human leukocyte antigen (HLA) haplotype DRB1*∗* 15 : 01 as a possible risk factor [20]. The researchers hypothesize that the presence of LN itself promotes the production of anti-GBM antibodies [20]. A common mechanism of anti-GBM positivity and LN remains elusive. However, kallikrein gene abnormalities have been speculated to play a role. Specifically, Lui et al. report that by antagonizing or stimulating mice with genetically altered kallikrein genes, they discovered altered susceptibility to anti-GBM-associated nephritis and spontaneous LN [6]. They suggest that kallikrein genes, which encode for serine esterases, are protective against anti-GBM-associated LN [6]. Li et al. report on sequencing and functional studies on the kallikrein gene cluster as associated with spontaneous LN and anti-GBM-induced GN [5]. Their study group subsequently used an adenoviral vector to deliver the kallikrein gene into a lupus-susceptibility interval on chromosome 7 and ameliorated anti-GBM-induced LN [5].

Our patient was anti-GBM-positive, and the first biopsy showed crescents in 2/4 glomeruli and granular to segmental deposits of IgG and C3 by immunofluorescence. These findings suggested a presumptive diagnosis of antiglomerular basement disease, consistent with our serological results, despite the lack of ultra-linear deposits of IgG. However, our second biopsy showed no crescents and no segmental immune complex deposition but granular deposits of IgG/IgA/IgM/C3/C1q/Kappa/Lambda (full-house pattern). Since there was endocapillary proliferation associated with spikes by light microscopy, the final diagnosis was consistent with combined focal proliferative and membranous lupus nephritis (III + V) [21]. Reconciling and finding a common pathophysiologic process to explain these disparate findings remains a challenge; however, research supports an interesting link between anti-GBM antibodies and MLN.

To directly study the relationship between anti-GBM and MLN, Wang et al. immunized six DBA/1, DBA/2, and WKY inbred strains of mice already susceptible to autoimmune glomerulonephritis with the main linear target of anti-GBM disease, *α*3 (IV) NC1 [22]. The investigators reported that after 8 weeks they measured maximum titers of circulating antibodies against the antigens, and 5/6 of the mice showed proteinuria at 8–10 weeks with granular deposition of IgG, C3, and C5b-9 and GBM thickening under electron microscopy, consistent with MGp [22]. These findings were recently published in the American Journal of Nephrology and provide a possible human biological mechanism linking anti-GBM-positive LN and MLN.

The two renal biopsies of this patient demonstrated a sequential transition from segmental deposits to granular deposits. Although no linear deposits were seen, these findings are consistent with reports of rare MGp due to anti-GBM antibodies [14], where linear to segmental anti-GBM deposits were eventually regrouped as granular deposits. An immunological understanding of this unusual MGp variant based on animal models is under investigation, but hypotheses explaining this sequential transition seen on biopsy include rearrangement of immune complexes (IC), the presence of unique target antigens within a genetically distinct chain of type IV collagen (COLIV alpha 3 NC1 domain), or a phenomenon called epitope spreading [14].

To facilitate experimental proof, Mannik et al. injected cross-linked and noncross-linked soluble antigen-antibody (NAP_19_-HAS-anti-NAP) complexes into female C57BL/6J mice. The mice were culled, and IF and EM were performed on renal tissue. Postinjection monitoring at early time points revealed IgG peritubular IF staining under both conditions [23]. However, after 4 hours, glomerular staining was seen only in the mice given the noncross-linked complexes. On EM, mice given noncross-linked complexes showed minimal electron dense deposits at 1 hour but extensive deposition after 4 hours whereas mice who received cross-linked complexes did not develop electron dense deposits after 4 hours [23]. The authors postulate that circulating ICs, after initial attachment to glomeruli, must undergo further rearrangement into larger deposits to persist as detectable electron dense deposits [23]. In our patient, this raises the possibility that over time, anti-GBM Ab associated ICs may undergo a similar transition from linear anti-GBM-associated pathology to granular deposition associated membranous nephropathy. During this temporal time delay immunological rearrangement may take place the result of which was seen on the sequential biopsy.

Class V Membranous LN has long been known to be associated with IC deposition in the subepithelial space, which leads to complement activation [24]. Injury to glomerular epithelial cells follows, with GBM thickening and the generation of nephrotic range proteinuria in the absence of inflammation due to podocyte foot effacement [23]. In regard to this rare variant of MGp involving anti-GBM antibodies, research shows the likely binding of ICs to the noncollagenous domain 1 (NC1) of *α*3 (IV) collagen (11–14) [25]. This was shown by Zhang et al. through use of an active mouse model of MG, where wild type mice immunized with rh-*α*3NC1 developed nephrotic range proteinuria, GBM thickening, IgG and C3 granular deposits on IF, and IC dense deposits on EM with podocyte foot process effacement [24]. The authors report how ICs associated with MGp may be caused by *α*3 (IV) GBM antibodies. They do not speculate on the precise underlying mechanism; however, epitope spreading may play a role.

Epitopes are specific peptide sequences on an antigenic molecule that are recognized by antibodies for binding [26]. Most antibodies bind to and react with numerous epitopes with different affinities, some of which are dominant. Some epitopes are theorized to require a conformational change in their molecular structure for direct antibody binding and the generation of an immune response. The activation of these secondary cryptogenic, nondominant epitopes via antibody binding is termed epitope spreading and may lead to a persistent immune response. Of note, these secondary epitope responses arise later than the principal response to the dominant epitope [26]. It has been theorized that the chronicity seen in many autoimmune diseases involves an initial immunological insult with persistence due to the de novo production of additional epitopes. This is likely a natural feature of our immune system since diversification is required to effectively attack and clear the maximum number of infections [26] and other insults. In our case, circulating anti-GBM antibodies may continually stimulate the upregulation and alteration of anti-GBM epitopes, leading to the development of this rare granular membranous nephritis variant. This is consistent with human studies on SLE [27] and bullous pemphigoid [28] and the use of animal models for murine relapsing experimental autoimmune encephalomyelitis (R-EAE) [29] and on nonobese diabetic mice with immune-mediated islet cell destruction [30]. In our case, anti-GBM antibodies may initially bind to the unique antigen subunit COLIV alpha 3 NC1 domain. MGp may then arise over time due to immune rearrangement or biochemical changes in the molecular structure of the anti-GBM epitope, leading to immune-mediated T- and B-cell activation and eventual glomerular MGp with heavy proteinuria. However, this remains speculative, and more research is needed.

## 4. Conclusion

LN can present with a wide variety of clinical and pathological manifestations within various demographic groups. We present a seronegative but anti-GBM-positive patient with combined focal and membranous lupus nephritis. The exact pathophysiology of this rare combination is unclear. Our biopsy results and animal research literature suggest that in a small subset of patients, anti-GBM antibodies may lead to the development of MGp due to immune rearrangement or epitope spreading. Certain individuals may be predisposed to this particular condition due to anomalies in the kallikrein gene.

## Data Availability

The data used to support the findings of this study are available from the corresponding author upon request.
